# Imaging spectrum of amyloid-related imaging abnormalities associated with aducanumab immunotherapy

**DOI:** 10.3389/fradi.2023.1305390

**Published:** 2024-01-05

**Authors:** Houman Sotoudeh, Mohammadreza Alizadeh, Ramin Shahidi, Parnian Shobeiri, Zahra Saadatpour, C. Austin Wheeler, Marissa Natelson Love, Manoj Tanwar

**Affiliations:** ^1^ Department of Radiology, University of Alabama at Birmingham, Birmingham, AL, United States; ^2^ Physiology Research Center, Iran University of Medical Sciences, Tehran, Iran; ^3^ School of Medicine, Bushehr University of Medical Sciences, Bushehr, Iran; ^4^ Department of Radiology, Memorial Sloan Kettering Cancer Center, New York, NY, United States; ^5^ Department of Neurology, University of Alabama at Birmingham, Birmingham, AL, United States

**Keywords:** Alzheimer’s disease, amyloid related imaging abnormalities, aducanumab, neuroimaging, anti amyloid aggregation

## Abstract

Alzheimer's Disease (AD) is a leading cause of morbidity. Management of AD has traditionally been aimed at symptom relief rather than disease modification. Recently, AD research has begun to shift focus towards disease-modifying therapies that can alter the progression of AD. In this context, a class of immunotherapy agents known as monoclonal antibodies target diverse cerebral amyloid-beta (Aβ) epitopes to inhibit disease progression. Aducanumab was authorized by the US Food and Drug Administration (FDA) to treat AD on June 7, 2021. Aducanumab has shown promising clinical and biomarker efficacy but is associated with amyloid-related imaging abnormalities (ARIA). Neuroradiologists play a critical role in diagnosing ARIA, necessitating familiarity with this condition. This pictorial review will appraise the radiologic presentation of ARIA in patients on aducanumab.

## Introduction

Alzheimer's Disease (AD), a neurodegenerative brain disorder, is a leading cause of morbidity in the world and is on the rise as the global population ages. From 2000 to 2019, patients affected by AD grew by more than 145%. Approximately sixty-five million Americans aged 65 and older are affected by AD, ranked as the sixth most common cause of mortality in the United States in 2019. The expense of care is rising in tandem. In 2022, it is anticipated that $321 billion will be spent to meet the healthcare and hospitalization needs of dementia patients aged 65 and older (1). While AD presents progressive deterioration in cognitive functions such as memory, judgment, and problem-solving, the exact pathophysiology remains unknown. The hypothesis of Aβ aggregation is one of the leading paradigms theorized. Amyloid precursor protein (APP) is consecutively cleaved by β-secretase and γ-secretase to form amyloid-beta (Aβ), and any mutation in this pathway can lead to aberrant Aβ formation. Extracellular Aβ aggregates and intracellular tau protein form intracellular neurofibrillary tangles (NFT), which lead to brain damage and synaptic loss in the memory-forming brain structures, including the hippocampus, cortex, and limbic system (2).

Most AD therapeutic research focuses on disease-modifying agents that will not alter the progression of AD (3). Gamma-secretase, beta-secretase inhibitors, and monoclonal antibodies aim to reduce the synthesis of neurotoxic Aβ (4). Monoclonal antibodies target diverse Aβ epitopes and exhibit distinct binding specificity, which can impede the disease's advancement and must be administered early in the course of the disease (5). Four anti-Aβ monoclonal antibodies are commonly used, including solanezumab, gantenerumab, aducanumab, and BAN2401. Few anti-Aβ medicines have been tested in clinical studies, and the results of clinical trials confirm that elevated Aβ is an early stage in AD progression. These trials have demonstrated significant gains by enrolling patients in secondary preventive trials to slow down cognitive decline during the preclinical stages of AD (6).

The Aβ-directed monoclonal antibody known as aducanumab targets the aggregated forms of Aβ, including soluble oligomers and insoluble fibrils (5). Aducanumab has shown promising clinical and biomarker efficacy; however, higher dosages of aducanumab demonstrate a risk of vasogenic edema, preventing greater doses from being administered (7). Amyloid-related imaging abnormalities (ARIA) were discovered to be the most notable safety finding, which was associated with Aβ elimination. These radiologic manifestations have been dose-dependent and more prevalent in apolipoprotein E (ApoE)-ɛ4 carriers. Aducanumab was authorized by the US Food and Drug Administration (FDA) to treat AD on June 7, 2021. Although the drug's approval was very controversial, aducanumab is the first disease-modifying medication for AD, which gives millions of patients hope (5). After an initial titration, the standard recommended dosage of aducanumab is 10 mg/kg. Aducanumab is administered intravenously over 1 h every four weeks and at least 21 days apart.

Magnetic resonance imaging (MRI) acquired during clinical trials of monoclonal antibodies targeting Aβ in AD patients has revealed amyloid-related imaging abnormality edema/effusion (ARIA-E) and amyloid-related imaging abnormality hemorrhage/hemosiderin deposition (ARIA-H) (8). Cerebral edema or sulcal effusion manifests as hyperintensity on fluid-attenuated inversion recovery (FLAIR) sequence that characterizes ARIA-E. The sulcal FLAIR hyperintensities indicate the presence of proteinaceous fluid within the subarachnoid or leptomeningeal space. The residual hemorrhage with hemosiderin deposition causes ARIA-H to manifest as focal, spherical, or linear signal abnormalities on the T2*-weighted gradient recalled echo (GRE) and Susceptibility weighted imaging (SWI) sequences (9).

While T2*-weighted GRE imaging continues to be the recommended approach in clinical trials, susceptibility-weighted imaging (SWI) is predominantly used by most centers across all vendors, owing to its superior spatial resolution. This technique is particularly effective in visualizing parenchymal microhemorrhages and superficial siderosis (10–12).

GRE/SWI and FLAIR sequences are the primary MR sequences used to diagnose and manage ARIA. The diffusion-weighted imaging (DWI) sequence is normal in ARIA, and it can be helpful to exclude cytotoxic edema/infarction as the cause of parenchymal FLAIR hypersignal intensities. FLAIR sequence is superior to T1 and T2 sequences in depicting the parenchymal signal alteration and sulci effusion. Although T1-weighted imaging typically has a limited role in these scenarios, it becomes more valuable in clinical trials when acquired using a 3D technique, enabling precise hippocampal volumetric calculations. Post-contrast T1 sequence usually shows no to minimal enhancement corresponding to the FLAIR signal alteration and does not be utilized for ARIA grading (13).

The clinical significance of ARIA varies with its intensity and involved location. ARIA-E may include concurrent symptoms such as headache, altered mental status, disorientation, gait difficulties, tremors, vomiting, or nausea (14) and may necessitate interventions beyond delaying medication (15).

ARIA-E and ARIA-H may be regionally or temporally connected, or they may be separately identified. In a bapineuzumab study, for instance, ARIA-H occurred in around 50% of people with ARIA-E, but not always concurrently and often before or after ARIA-E (16). Both ARIA-E and ARIA-H manifest due to the same mechanism related to increased vascular permeability/leakage; however, the appearance of an imaging abnormality depends on the composition of leaked products. Proteinaceous fluid is also believed to escape whenever red blood cells rupture, such that ARIA-E occurs to a certain degree with any ARIA-H. ARIA-E is temporary and resolves within weeks to months, but the hemosiderin deposition from ARIA-H often does not resolve (17). Treatment-related ARIA-H will frequently be the precursor of modest ARIA-E. In contrast, ARIA-E may develop even in the absence of ARIA-H. In conclusion, it is probable that the timing of imaging in relation to the time of vascular leak accounts for the differential detection of ARIA-H and ARIA-E.

Although largely asymptomatic, ARIA typically occurs early in treatment, often within weeks or months, varying by drug and dosage. Monitoring for new symptoms is essential, especially after initial treatments or at the highest doses. Regular MRI surveillance is advised to prevent severe ARIA. For aducanumab, MRIs are recommended within one year before treatment initiation and before the 7th (10 mg/kg) and 12th infusions. Despite dose adjustments and monitoring, the majority of ARIA cases still occur within the initial eight-dose period, presumably linked to a temporary phase of compromised vessel wall integrity. Further, the current guidelines suggest additional MRIs at the 5th (6 mg/kg) infusion and 10th dose for ApoE4 carriers (18, 19). Following the twelfth infusion, it is proposed that imaging examinations be conducted at intervals of 6–12 months for a period of up to five years (20).

If a patient develops ARIA symptoms, clinical evaluation should be performed, as well as a brain MRI. In case of positive symptoms and ARIA findings, the clinicians must reevaluate the patient and consider dose reduction or treatment termination if symptoms are severe. ARIA-E is often self-limited and most of the patients can resume the treatment after a temporary treatment suspension with or without steroid therapy. On the other hand, the patients with “severe” ARIA-H (more than ten cerebral microhemorrhages, and cerebral hematoma larger than 1 centimeter) are not eligible to resume treatment (21).

The neuroradiologist is advised to adopt a standardized lexicon for reporting, as delineated in Supplementary Table 1. This grading system is based on visual evaluation of brain MR images and comparing the follow-up and baseline MRIs. Presence, numbers and the size of A. New paranchymal FLAIR hypersignal intensities, B. New Sulci FLAIR hypersignal intensities and C. New microhemorrhages/superficial siderosis can determine the severity of ARIA. This correlation suggests a feasible alignment of treatment management protocols across these scales to standardize neuroradiological assessments (22).

Notably, indistinguishable ARIA clinical processes and radiological alterations appear in cerebral amyloid angiopathy (CAA)–related inflammation (CAA-ri). This condition is an autoimmune brain disorder characterized by elevated levels of autoantibodies in the cerebrospinal fluid (CSF). These autoantibodies specifically target the Aβ protein that accumulates in the walls of the brain's cortical and leptomeningeal arteries, arterioles, and capillaries. CAA-RI is marked by the sudden or gradual onset of neurological symptoms ranging from mild cognitive disturbances and headaches to rapidly worsening cognitive impairment, seizures, changes in mental state, and specific neurological deficits. Nonetheless, the neurological symptoms of CAA-RI show remarkably similar neuroimaging findings to those seen in ARIA (23). This similarity and other conditions that need to be differentiated from ARIA are discussed in Table 1. Given the current guideline, the CAA patients are not eligible for aducanumab treatment (23).

**Table 1:** Differential diagnoses of ARIA.

Differential diagnosis	Similarity to ARIA-E	Similarity to ARIA-H	Differentiation
CAA-ri	Unifocal or multifocal areas of subcortical vasogenic edema with mild mass effect superimposed on a background of CAA/White matter hyperintensity (23)/imaging features suggesting noncytotoxic edema on DWI sequences (13)	Microhemorrhages, siderosis, and chronic parenchymal hematoma) (23)/gradient recalled-echo/T2*-weighted images may show associated foci of parenchymal microhemorrhages (13)	Clinical history of monoclonal antibodies administration (23)
PRES	Predilection for the occipital lobes with petechial hemorrhages/reversible T2 hyperintensities (24–26)	Edema and microhemohrages	While most ARIA cases are asymptomatic, PRES typically manifests with significant symptoms. History of hypertension, preeclampsia, and medications can favor PRES (9)
Subarachnoid hemorrhage	Leptomeningeal FLAIR hyperintensity (13)	Sulci GRE or SWI signal void intensities	CT and lumbar puncture to exclude subarachnoid hemorrhage (13)
Inadequate CSF-nulling artifacts	Leptomeningeal FLAIR hyperintensity (13)		Centering the patient in the receive coil can result in less artifactually hyperintense areas/loading the coil correctly/Using the proper size coil/Shimming to reduce inhomogeneity of the magnetic field (19). Lack of sulcu FLAIR signal suppression can be secondary to oxygen supplement.
Meningitis	Leptomeningeal FLAIR hyperintensity (13)		Clincal presentation (19).
Ischemia	Parenchymal FLAIR hyperintensity (13)		DWI can evaluate infarction with cytotoxic edema (13)
Neoplasm/metastatic disease	Parenchymal FLAIR hyperintensity (13)		Gadolinium administration can exclude malignancy (13)
Cerebritis	Parenchymal FLAIR hyperintensity (13)		Gadolinium administration can demonstrate cerebritis and brain abscesses (13)

ARIA-E, amyloid-related imaging abnormalities suggestive of vasogenic edema; ARIA-H, amyloid-related imaging abnormalities indicative of hemosiderin deposition; CAA-ri, cerebral amyloid angiopathy-related inflammation; CSF, cerebrospinal fluid; CT, computed tomography; DWI, diffusion weighted imaging; FLAIR, fluid-attenuated inversion recovery; GRE, gradient-recalled echo; PRES, posterior reversible encephalopathy syndrome; SWI, susceptibility weighted imaging.

## Clinical examples

**Figure 1:**
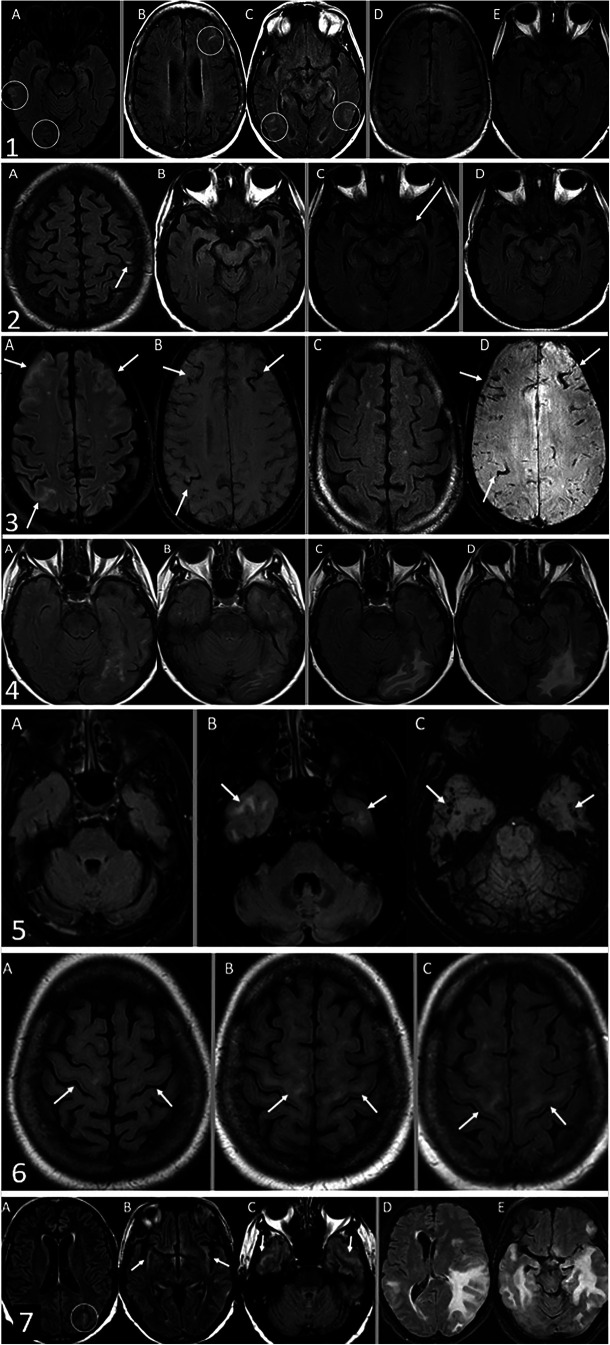
Case 1. ARIA-E with interval resolution. The baseline MRI did not demonstrate any acute findings, only chronic atrophy and microvascular angiopathy (not shown). The safety MRI by FLAIR sequence (**A**) 42 days after baseline MRI revealed interval development of sulcal FLAIR hyperintensities compatible with sulcal effusions in the right temporal and occipital lobes. Thirty-two days later, the first follow-up MRI (B and C) revealed new sulcal effusions in the left frontal, left posterior temporal, and right temporal-occipital lobes. Subsequent MRI after another 58 days (D and E) demonstrated complete interval resolution of these sulcal effusions. Case 2. ARIA-E. The safety MRI by FLAIR sequence (**A**) revealed interval development of a tiny sulcal effusion in the left frontal lobe, compatible with ARIA-E. Of note, the medial temporal lobes were normal (**B**) 26 days later, the first follow-up MRI (**C**) revealed a new subtle FLAIR hyperintensity in the medial left temporal lobe. Subsequent MRI after another 31 days (**D**) demonstrated interval resolution of FLAIR hyperintensity in the medial left temporal lobe. Case 3. ARIA-E and H. The safety MRI (A: FLAIR sequence and B: SWI sequence) revealed interval development of sulcal effusions in the bilateral frontal and right parietal lobes. Trace subarachnoid hemorrhages are also evident at the same locations (**B**) Follow-up MRI after 70 days (C: FLAIR sequence and D: SWI sequence) demonstrated interval resolution of sulcal effusions but persistent sulcal hemosiderin deposition (**D**) Case 4. ARIA-E by FLAIR sequence. The safety MRI (A and B) revealed interval development of vasogenic edema in the left occipital lobe. Follow-up MRI after 30 days(C and D) demonstrated worsening edema. Case 5. ARIA-E and H. A baseline FLAIR sequence (A: FLAIR sequence) is unremarkable. The safety MRI revealed interval development of vasogenic edema (B: FLAIR sequence) and microhemorrhages (C: SWI sequence) in the bilateral anterior temporal lobes. Case 6. ARIA-E with FLAIR hyperintensity involving the subcortical corticospinal tracts as a manifestation of ARIA. Baseline MRI (**A**) demonstrated normal signal intensity of subcortical corticospinal tracts. The first follow-up MRI after 98 days revealed interval development of symmetrical FLAIR hyperintensities in the subcortical corticospinal tracts (**B**), which remain unchanged on subsequent MRI obtained three months later (**C**) Case 7. Severe ARIA-E by FLAIR sequence. The safety MRI (A-C) revealed trace sulcal effusion in the left occipital lobe (**A**) and minimal FLAIR hyperintensities in the bilateral insular cortices and anterior temporal lobes (B and C). Seven months later, the patient presented with behavior and cognition changes as well as nighttime confusion with hallucinations. A follow-up MRI performed at that time (D and E) demonstrated marked worsening of multifocal edema of the temporal lobes with new foci in the left frontal lobe, associated mass effect, effacement of left lateral ventricle, new left-to-right midline shift, and left uncal herniation.

We present our experience with ARIA-E and ARIA-H observed at our tertiary care hospital, a participating site for a multicenter aducanumab trial. Baseline and follow-up MR images of all of these patients were reviewed by two neuroradiologists (15 and 3 years of experience after graduation). Seven cases are being presented here to review the classical presentation of ARIA on MRI (Figure 1).

## Conclusion

Recent Alzheimer's disease-modifying drug trials have led to new opportunities and challenges for neuroradiologists, who play a critical role in the appropriate selection and follow-up of these patients. It is critical to recognize radiologic patterns of ARIA and be familiar with common time intervals of occurrence and expected evolution. Radiologists play a critical role in ensuring the safety of patients undergoing these trials.
