# Supplementary material for: Imaging spectrum of amyloid-related imaging abnormalities associated with aducanumab immunotherapy
Source: Front Radiol. 2024 Jan 5;3:1305390. doi: 10.3389/fradi.2023.1305390 (PMC10796528; doi:10.3389/fradi.2023.1305390)
Supplement: Supplementary file 1 [file Table1.docx]

**Table 1: ARIA MRI Severity Criteria**

| **ARIA Type** |  | **Radiographic Severity** |  |
| --- | --- | --- | --- |
|  | **Mild** | **Moderate** | **Severe** |
| ARIA-E | Sulcal, cortical or subcortical FLAIR hyperintensity in 1 location and < 5 cm in size | FLAIR hyperintensity between 5 to 10 cm, OR  more than 1 location of involvement, each measuring < 10 cm | FLAIR hyperintensity > 10 cm in size, often with significant subcortical white matter and/or sulcal involvement |
| ARIA-H microhemorrhage | Less than 4 new cerebral microhemorrhages | Between 5 to 9 new cerebral microhemorrhages | 10 or more new cerebral microhemorrhages |
| ARIA-H superficial siderosis | 1 focal area of superficial siderosis | 2 focal areas of superficial siderosis | More than 2 focal areas of superficial siderosis |
